# Binding structures of SERF1a with NT17-polyQ peptides of huntingtin exon 1 revealed by SEC-SWAXS, NMR and molecular simulation

**DOI:** 10.1107/S2052252524006341

**Published:** 2024-08-08

**Authors:** Tien-Chang Lin, Orion Shih, Tien-Ying Tsai, Yi-Qi Yeh, Kuei-Fen Liao, Bradley W. Mansel, Ying-Jen Shiu, Chi-Fon Chang, An-Chung Su, Yun-Ru Chen, U-Ser Jeng

**Affiliations:** a Department of Chemical Engineering National Tsing Hua University Hsinchu300044 Taiwan; b National Synchrotron Radiation Research Center Hsinchu300092 Taiwan; c Genomics Research Center Academia Sinica Taipei115024 Taiwan; d Chemical Biology and Molecular Biophysics Program, Taiwan International Graduate Program, Institute of Biological Chemistry, Academia Sinica, Taipei115024, Taiwan; e Institute of Biochemical Sciences National Taiwan University Taipei106319 Taiwan; f College of Semiconductor Research National Tsing Hua University Hsinchu300044 Taiwan; SPring-8, Japan

**Keywords:** huntingtin exon 1, polyglutamine peptides, SERF1a, SEC-SWAXS, NMR, molecular simulation, Huntington’s disease

## Abstract

The aberrant fibrillization of huntingtin exon 1 (Httex1) characterized by an expanded polyglutamine (polyQ) tract is a defining feature of Huntington’s disease, a neurodegenerative disorder. Recent investigations underscore the involvement of a small EDRK-rich factor 1a (SERF1a) in promoting Httex1 fibrillization through interactions with its N terminus. By establishing an integrated approach with size-exclusion-column-based small- and wide-angle X-ray scattering (SEC-SWAXS), NMR, and molecular simulations using *Rosetta*, the analysis here reveals a tight binding of two NT17 fragments of Httex1 (comprising the initial 17 amino acids at the N terminus) to the N-terminal region of SERF1a. In contrast, examination of the complex structure of SERF1a with a coiled NT17-polyQ peptide (33 amino acids in total) indicates sparse contacts of the NT17 and polyQ segments with the N-terminal side of SERF1a. Furthermore, the integrated SEC-SWAXS and molecular-simulation analysis suggests that the coiled NT17 segment can transform into a helical conformation when associated with a polyQ segment exhibiting high helical content. Intriguingly, NT17-polyQ peptides with enhanced secondary structures display diminished interactions with SERF1a. This insight into the conformation-dependent binding of NT17 provides clues to a catalytic association mechanism underlying SERF1a’s facilitation of Httext1 fibrillization.

## Abstract 2

Binding structures of SERF1a with the N-terminal fragment of huntingtin exon 1 and NT17-polyQ peptides are revealed using an integrated analysis of size-exclusion-column-based small- and wide-angle X-ray scattering (SEC-SWAXS), NMR, and molecular simulation.

## 1. Introduction

Huntington’s disease, a neurodegenerative disorder, is caused by CAG (cytosine, adenine, guanine) expansion in exon 1 of the huntingtin (Htt) gene, leading to progressive brain dysfunction with signs of psychiatric disorder, muscle coordination impairment and cognitive decline (Matlahov & van der Wel, 2019 ▸). The abnormal expansion translates to mutant Htt proteins (huntingtin exon 1 or Httex1) characterized by an expanded polyglutamine (polyQ) (more than 35–40 glutamine) tract flanked by N-terminal 17 amino acids (NT17) and a C-terminal polyproline domain. The polyQ segments are prone to misfold and form α-helical packing. Subsequently, the helical packing converts to β-sheets for fibrillization conversion and deposition on neurons, leading to neuro­degenerative diseases (Arndt *et al.*, 2015 ▸). Previous studies showed that the amyloid fibrillization process could be enhanced by human small EDRK-rich factor 1a (SERF1a), through α-helix interactions with the NT17 domain of Httex1 (Tsai *et al.*, 2023 ▸; Falsone *et al.*, 2012 ▸). The interaction was revealed mainly from NMR observation that the α-helical regions of SERF1a were significantly influenced upon binding with Httex1-39Q protein (Tsai *et al.*, 2023 ▸). Moreover, the binding ratio of SERF1a with the synthesized NT17 peptide, simulating the N-terminal first 17 amino acids of Httex 1, was shown to be 1:2 on the basis of isothermal titration calorimetry (ITC) results. The binding was found to be weakened when NT17 was attached to α-helical polyQ. Alterations in the radius of gyration (*R*_g_) of SERF1a were noted following its binding with NT17 and with NT17-polyQ, as observed through small-angle X-ray scattering (SAXS) analysis (Tsai *et al.*, 2023 ▸). Although the NMR and SAXS data have provided sufficient evidence and structural features of SERF1a–polyQ binding, the specific molecular structures that could better characterize this binding mechanism remain unresolved. A deeper understanding of how the polyQ segment structure influences the binding of NT17-polyQ peptides to SERF1a continues to be a significant area of interest.

Probing solution binding structures of the complex of SERF1a and NT17-polyQ peptides could benefit greatly from integrated methodologies of complementary structural sensitivities. Recent reviews (Schroer & Svergun, 2018 ▸; Jeffries *et al.*, 2021 ▸; Bizien *et al.*, 2016 ▸; Trewhella *et al.*, 2017 ▸) illustrate a development trend of SAXS with online size-exclusion chromatography (SEC) for complex conformations of biomolecules in solution. Further incorporated with optical measurements of UV–Vis absorption and differential refractive index (dRI) (Blanchet *et al.*, 2015 ▸), SEC-SAXS can reveal conformation and composition of a biomolecular binding complex in one sample elution (Shih *et al.*, 2023 ▸, 2017 ▸; Yeh *et al.*, 2017 ▸). Very recently, SEC-SAXS was further extended to wide-angle X-ray scattering (WAXS) for SEC-SWAXS, allowing better model discrimination and molecular model construction (Shih *et al.*, 2022 ▸; Bizien *et al.*, 2016 ▸; Schroer & Svergun, 2018 ▸). With SEC-SWAXS structural sensitivity, hydration water structures near the surface of biomolecules become relevant in the SWAXS data analysis, as demonstrated recently (Hub, 2018 ▸; Manalastas-Cantos *et al.*, 2021 ▸).

In this study, we establish a combined analysis of SEC-SWAXS, NMR and molecular simulation to construct the molecular models of SERF1a, NT17, NT17-polyQ peptides and their complexes in solution. The UV–Vis absorption and dRI data concomitantly measured along the SEC-SWAXS sample elution provide complementary the composition information, which is crucial in the constructions of the complex structures. These constructed molecular structures provide hints to the association mechanism of SERF1a with the NT17-polyQ peptides.

## 2. Materials and methods

### 2.1. Sample preparation

SERF1a was purified from the human SERF1a gene as reported previously (Tsai *et al.*, 2023 ▸). NT17 containing the N-terminal first 17 amino acids of the Httex1 protein and NT17-polyQ peptides with 33 amino acids of Htt-0, Htt-1 and Htt-3 (*cf*. Table 1 ▸) were synthesized by the peptide synthesis core in Genomics Research Center, Academia Sinica. Htt-0 is of 14 successive glutamine amino acids attached to NT17, simulating the native polyQ structure of successive glutamines. Htt-1 has four leucine residues replacing residues of position *a*/*d* in the polyQ region of Htt-0, to enhance α-helix formation (Fiumara *et al.*, 2010 ▸); whereas, for Htt-3, the positions *a*/*d* in the polyQ region are substituted by four prolines to suppress formation of α-helix structure (Table 1 ▸, see italic letters for the replaced residues). Sample solutions of SERF1a (4.1 mg ml^−1^), NT17 (1.6 mg ml^−1^) and Htt-0, Htt-1 and Htt-3 (all of 10 mg ml^−1^) were prepared with sodium phosphate buffer (PB) solution (containing 480 µl of 10 m*M* PB, pH 7.4, 16.5 µl of 100 m*M* NaOH and 10 µl of 1% TFA). To avoid sedimentation due to strong binding, concentration-reduced solutions of 0.255 mg ml^−1^ SERF1a (61 µ*M*) and of 0.445 mg ml^−1^ NT17 (129 µ*M*) were prepared individually then mixed for a SERF1a:NT17 mixture of 1:2 molar ratio. A mixture of SERF1a:Htt3 with 1:2 molar ratio was prepared from sample solutions of 0.7 mg ml^−1^ SERF1a and 0.8 mg ml^−1^ Htt-3. All the sample solutions were filtered (0.22 µm pore size) prior to SWAXS measurements.

### 2.2. SEC-SWAXS

SWAXS data were measured at the Taiwan Photon Source (TPS) 13A BioSWAXS endstation of the National Synchrotron Radiation Research Center (NSRRC), using a 15 keV X-ray beam and two in-vacuum Eiger X 9M and 1M detectors for simultaneous SAXS and WAXS measurements, as detailed in previous reports (Shih *et al.*, 2022 ▸; Liu *et al.*, 2021 ▸). Sample solutions of 50–100 µl were injected into the SEC-SWAXS system, coupled with an in-line high-performance liquid chromatography (HPLC) unit (Agilent 1260 series) and UV–Vis absorption/refractive index spectrometers. With a flow rate of 0.35 ml min^−1^, the sample solution (sandwiched by the buffer solution, with a dilution factor of *ca*. 2–4) was directed to a quartz capillary (of 2 mm diameter and a wall thickness of 10 µm) thermostated at 283 K for simultaneous X-ray and UV–Vis exposures, followed by dRI measurements at *ca*. 40 cm downstream. Elution peaks and peak widths of the measured UV–Vis and dRI profiles were aligned through a profile broadening and alignment process by the software package *VISION* (WYATT Technology Corporation, Santa Barbara, California, USA) (including *VOYAGER* and *ASTRA*) for determination of the complex compositions. SWAXS data were collected continuously with 2 s per data frame over the elution peak. With the *TPS 13A SWAXS Data Reduction Kit* (*DRK*) Version 3.6, the frame data of well overlapped SAXS profiles were averaged and subtracted with buffer scattering (measured before or after the elution peak), followed by normalizations of the incoming X-ray flux and sample thickness, then rescaled to absolute scattering intensity in units of cm^−1^ via comparing with the absolute scattering intensity of water. All the presented SAXS data are not normalized by sample concentrations. Each frame of the elution SAXS data was routinely examined by our own data-evaluation package, which incorporates several functions of *ATSAS*, including (1) checking the Guinier region quality with *AUTORG*, (2) examining the Kratky plot for background-subtraction quality and structural compactness, (3) overlapping the selected SAXS data frames along the elution for a lack of concentration-dependency (interparticle interaction) check, and (4) generating distance distribution functions *p*(*r*) and executing *DAMMIF* for shape evaluation with an *ab initio* model. All these were detailed in our previous report (Shih *et al.*, 2022 ▸). The WAXS data frames corresponding to these processed SAXS data were then processed by the *DRK*. The two sets of data were merged for integrated SWAXS data, covering a wide scattering vector *q* range over 1.0 Å^−1^, where *q* = 4πλ^−1^sin θ is defined by the X-ray wavelength λ and scattering angle 2θ.

### 2.3. SWAXS data analysis with *I-TASSER*, *CRYSOL* and *Rosetta-fastsaxs*

Five initial three-dimensional atomic models with relatively low free energy of SERF1a, NT17 or the polyQ peptides were constructed using the *I-TASSER* protein structure and function prediction server (Roy *et al.*, 2010 ▸; Yang & Zhang, 2015*a* ▸,*b* ▸; Zhang, 2008 ▸), from multiple threading alignments and iterative structural-assembly simulations. The initial structures were built from scratch by *ab initio* modeling (Wu *et al.*, 2007 ▸), as the PDB library had no structurally related homologues for the coiled SERF1a and NT17-polyQ peptides. Subsequently, theoretical SWAXS profiles of the five output models were computed using the all-atom-calculation program *CRYSOL* (Svergun *et al.*, 1995 ▸), which fits the model-calculated SWAXS profiles to the experimental data using only two parameters of the average displaced solvent volume per atomic group and the contrast of a hydration layer. The models with reasonably low least-square fitting (χ^2^, goodness of fit) were selected for further energy minimization with SAXS data fitting χ^2^ using the *Rosetta* modeling suite (Bender *et al.*, 2016 ▸; Das & Baker, 2008 ▸; Kaufmann *et al.*, 2010 ▸), with a two-step free-energy minimization of backbones followed by side chains. The SAXS data fitting constraint was applied by adding the *fastsaxs* term in the *Rosetta* default scoring function *Talaris2014* (O’Meara *et al.*, 2015 ▸; Stovgaard *et al.*, 2010 ▸). The *fastsaxs* term contributes to the total *Rosetta* score as an effective energy score and was scaled from the SWAXS data fitting χ^2^ (up to *q* = 0.7 Å^−1^) by a weighting factor. The weighting factor of χ^2^ was set between 1 and 500 according to the scores of the other free-energy terms, to ensure that a low χ^2^ value below *ca*. 2–3 could be reached in the model search with the total *Rosetta* score minimization process (χ^2^ of SWAXS data fitting in a wider *q* range is generally higher than that of the SAXS data fitting alone). In the structure-optimization process of SERF1a, the NMR-determined α-helix and relevant residue structures were used as the model constraint. Each optimized model of SERF1a, NT17 or NT17-polyQ is a representative one selected from the ten best models of independent runs of the *Rosetta-fastsaxs* modeling. The degree of model convergence is represented by the normalized spatial discrepancy (NSD) of the three-dimensional points of the ten models, as a quantitative measure of conformation similarity; NSD = 0 indicates perfectly overlapped models, whereas NSD < 1 is a general criterion for converged resembled models (Konarev *et al.*, 2016 ▸; Prior *et al.*, 2020 ▸).

### 2.4. Composition determination of the complex

The number density *n*_o_ of a two-component complex of SERF1a (A) with NT17 (B) (or NT17-polyQ peptides) in solution with complexing numbers *N*_A_ and *N*_B_ can be determined from the SEC-SWAXS zero-angle scattering intensity *I*_0-SAXS_ (or *I*_0_), together with the sample concentrations determined from the simultaneously measured optical density *I*_UV_ of UV–Vis absorption and differential refractive index Δ*n*_dRI_, over the sample elution region (Shih *et al.*, 2018 ▸, 2023 ▸; Lin *et al.*, 2009 ▸). The three parameters of *n*_o_, *N*_A_ and *N*_B_ can be resolved analytically using the following set of equations:
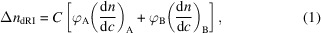

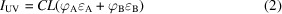
and
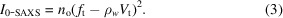
In equation (1), *C* is the weight concentration of the complex in solution, and the mass fractions of components A and B of the complex are φ_A_ = *N*_A_*M*_A_/*M*_t_ and φ_B_ = *N*_B_*M*_B_/*M*_t_, with the complex molar weight *M*_t_ = *N*_A_*M*_A_ + *N*_B_*M*_B_; (d*n*/d*c*)_A_ and (d*n*/d*c*)_B_ are the corresponding specific refractive index increments, and ɛ_A_ and ɛ_B_ are the UV–Vis light extinction coefficients. *N*_B_/*N*_A_ defines the association ratio χ_BA_. In equation (2), *L* is the sample path length defined by the sample capillary diameter. We note that *I*_0-SAXS_ was extrapolated from the SAXS data using the Guinier approximation, with data in the *q* range of *qR*_g_ < 1.3. In equation (3), *n*_o_ = *CN*_A0_/*M*_t_, where *N*_A0_ is Avogadro’s number and ρ_*w*_ is the scattering length density of the buffer. The total X-ray scattering length *f*_t_ = (*N*_A_*E*_A_ + *N*_B_*E*_B_)*f*_e_ is contributed to by the number of electrons *E*_A_ and *E*_B_ of A and B with the X-ray scattering length of electrons *f*_e_ = 2.8179 × 10^−5^ Å. The complex volume *V*_t_ = *N*_A_*V*_A_ + *N*_B_*V*_B_ is contributed to by two components, with *V*_A_ = 9719 Å^3^ of SERF1a and *V*_B_ = 3190 Å^3^ of NT17 calculated on the basis of their sequences (Jacrot, 1976 ▸). The d*n*/d*c* values measured individually for SERF1a and NT17 are 0.1847 and 0.1870 ml g^−1^, respectively; the corresponding UV–Vis molar absorption coefficients ε_214_ at 214 nm are 64 310 and 27 715 *M*^−1^cm^−1^, respectively (214 nm data were used for the UV–Vis absorption as the commonly used 280 nm is low for either NT17 or SERF1a). For Htt-0, Htt-1 and Htt-3, ε_214_ values are 49887, 49499 and 60019 *M*^−1^cm^−1^, respectively, as summarized in Table S1 of the supporting information.

## 3. Results and discussion

### 3.1. SERF1a

**Figure 1:**
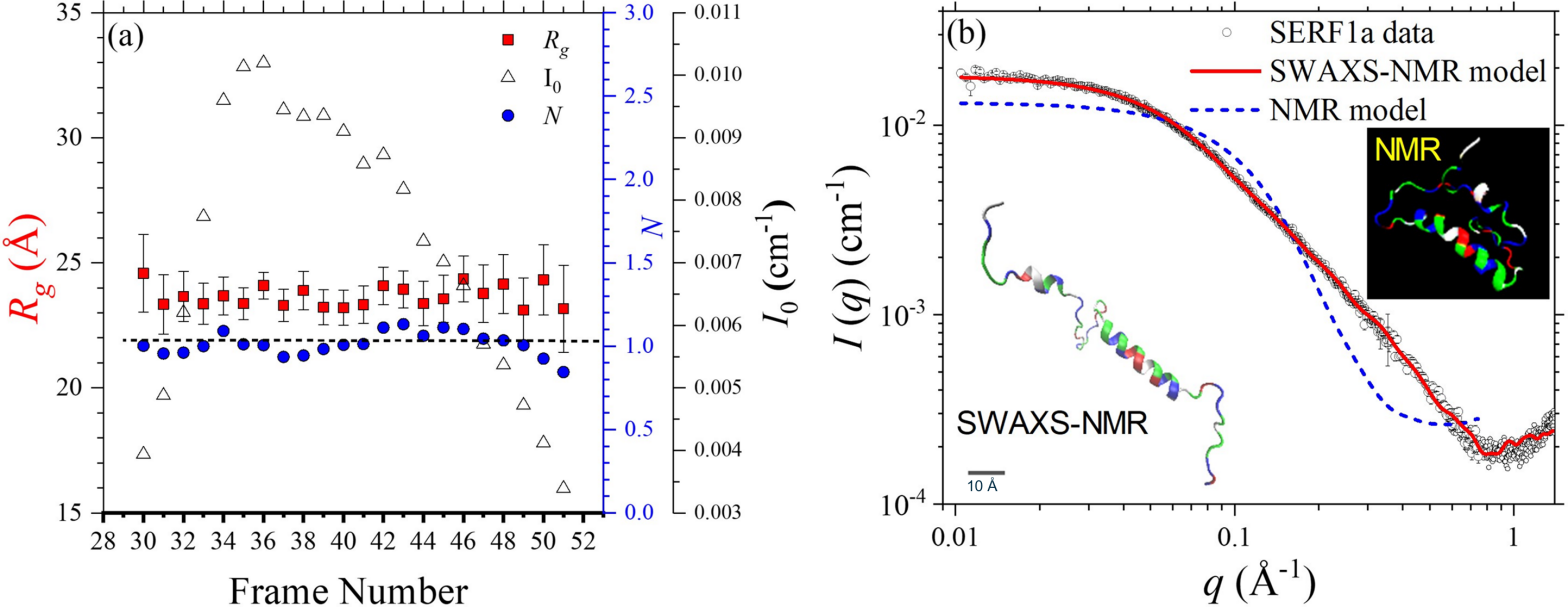
(*a*) Elution profiles of *R*_g_, zero-angle intensity *I*_0_ and non-aggregation number values near *N* = 1, deduced from the integrated analysis of SAXS *I*_0_ and optical data of SERF1a. (*b*) SWAXS data of SERF1a are fitted using an NMR model (inset) and the SWAXS-NMR model (inset, scaled with a bar for 10 Å size). The data and model are deposited in the SASBDB database with the code SASDVL5 (Kikhney *et al.*, 2020 ▸)

Fig. 1 ▸(*a*) shows SEC-SWAXS elution profiles of radius of gyration *R*_g_, zero-angle scattering intensity *I*_0_ and deduced aggregation number *N* of SERF1a, revealing largely monodisperse monomers (*N* = 1) of *R*_g_ = 23.5 ± 1.0 Å. The SWAXS data are compared with a theoretical profile [Fig. 1 ▸(*b*)] calculated from a SERF1a pure NMR structure model. The NMR structure model was selected from 10 000 models built from *Chemical-Shift-Rosetta* (*CS-Rosetta*) (Lange *et al.*, 2012 ▸) using the NMR backbone chemical shift data reported previously (Tsai *et al.*, 2023 ▸), on the basis of a lowest free energy. The apparent deviation of the calculated model profile from the SWAXS data in the low-*q* region indicates a deficiency of the model constructed merely from considerations of the NMR backbone structure with energy minimization. Subsequently, we used the NMR-derived model as an initial structure in the *Rosetta-fastsaxs* modeling. Whereby, all the NMR-determined helical segments (*cf*. Table 1 ▸) and relevant amino residues were used as the modeling constraints. As a result, an optimize model with a relatively elongated conformation incorporating all of the NMR-determined helical structure can fit nearly the full *q* range of the SWAXS data (Fig. 1 ▸). This model is representative of one of the ten best-fitted models obtained from ten independent runs of the *Rosetta-fastsaxs* modeling, with comparable free-energy scores and χ^2^ values as shown in Fig. S1 of the supporting information. The modest NSD value of 1.3, calculated from ten models with the same NMR-determined helical features and similar *R*_g_ values, suggests that SERF1a may exhibit a relatively converged conformation, despite some variability in the deployment of local non-rigid segments. In our previous report (Tsai *et al.*, 2023 ▸), we analyzed SAXS data of SERF1a using *Rosetta-fastsaxs* without NMR constraints. However, the model [shown in Fig. 1 ▸(*b*)] constructed from the SWAXS data with NMR constraints now provides a more refined orientation of SERF1a’s segmented helices.

### 3.2. NT17 conformation and transition via polyQ

**Figure 2:**
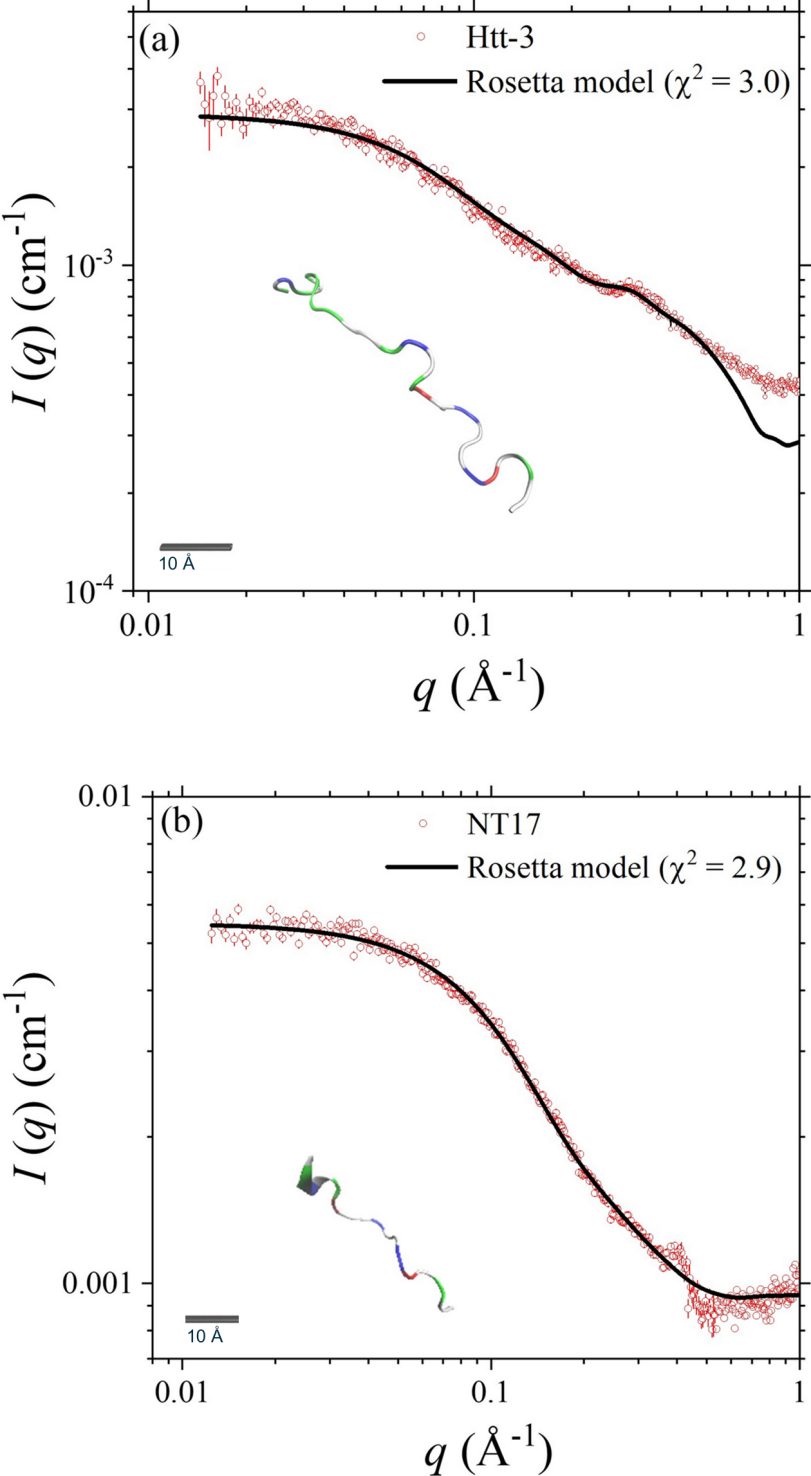
SWAXS data of (*a*) Htt-3 (SASDVM5) and (*b*) NT17 (SASDVN5) are fitted using the represented models shown in the insets (with a scale bar of 10 Å). The corresponding NSD values calculated from ten *Rosetta* models of repeated runs are 0.18 for Htt-3 and 0 for NT17.

Shown in Fig. 2 ▸(*a*) are the SWAXS data for the NT17-polyQ peptide Htt-3, with *R*_g_ = 16.7 Å extracted from the Guinier approximation. On the basis of the model searching process of *I-TASSER* and *CRYSOL*, followed by *Rosetta-fastsaxs* detailed above, a coil model (shown as an inset in the figure plot) was built to adequately fit the SWAXS data. This coil feature is consistent with that reported previously for a similar NT17-polyQ peptide at pH = 7 on the basis of NMR data analysis (Baias *et al.*, 2017 ▸). Furthermore, a parallel SWAXS data analysis for the segment of NT17 [Fig. 2 ▸(*b*)] consistently reveals a loose NT17 coil of *R*_g_ = 11.6 ± 0.5 Å. The revealed coil structures of NT17 and Htt-3 are consistent with their circular-dichroism (CD) spectra, *i.e.* both with no pronounced secondary structure measured (Fig. S2).

**Figure 3:**
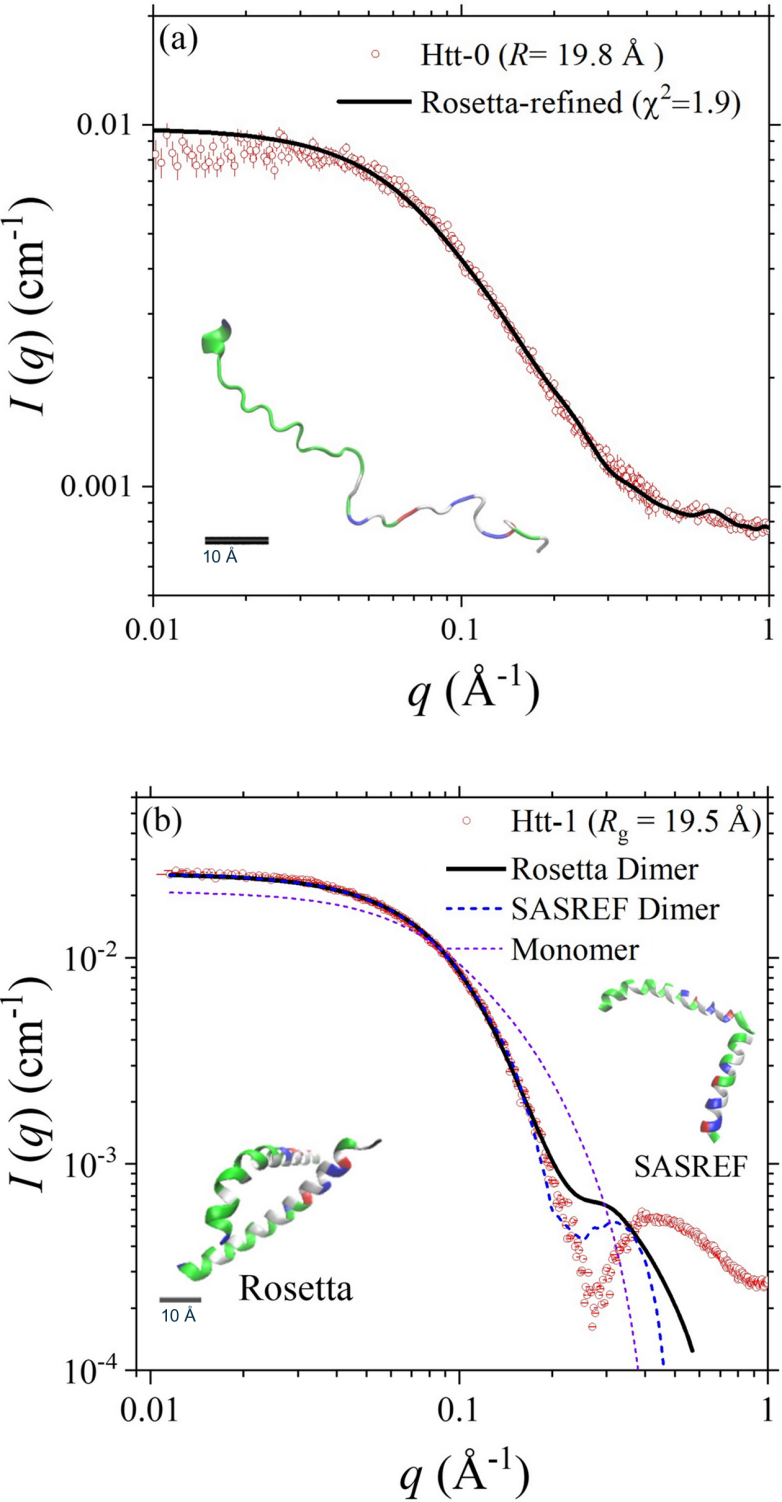
(*a*) SWAXS data of Htt-0 (SASDVP5) are fitted using the *Rosetta* model shown in the inset (the scale bar in the inset is for 10 Å). (*b*) SWAXS data of Htt-1SASDVQ5 are fitted using the dimer models optimized by *Rosetta-fastsaxs* and *SASREF*, as shown in the insets. Also shown is the profile calculated with a helical monomer model of Htt-1.

In contrast, SWAXS data analysis for Htt-0 [Fig. 3 ▸(*a*)] indicates that the polyQ region of 14 successive glutamine amino acids attached to NT17 could form a more extended structure, with an *R*_g_ value of 19.8 Å significantly larger than that (16.7 Å) of the coiled Htt-3. The slightly richer helical feature of the *Rosetta-fastsaxs* model is consistent with the higher α-helical content revealed from the CD spectrum measured (Fig. S2). In the case of Htt-1 of a specially designed polyQ sequence with leucines for enhancing α-helical structure, the CD spectrum indeed reveals a dominant α-helical feature (Fig. S2); our molecular-simulation result also consistently suggests a highly helical structure of Htt-1, including the NT17 and the helical-enhancing polyQ regions. Namely, NT17 could be directed by the helical polyQ segment to form a helical conformation. The SWAXS data measured [Fig. 3 ▸(*b*)] reveal a dimer conformation for Htt-1, as determined from the zero-angle scattering intensity *I*_0_ value and the sample concentration measured from UV–Vis absorption. We, therefore, fitted the SWAXS data with two helical Htt-1 placed close to each other as an initial model. As a result, the best dimer conformation shown in Fig. 3 ▸(*b*) can largely fit the data in the lower-*q* region, supporting the dimer conformation. Nevertheless, the local structures of the dimer model could not adequately describe the broad hump centered at *q* ≃ 0.45 Å^−1^; this hump is attributed to a feature of helix–helix correlation of the two Htt-1 in a dimer form that could not be observed in the SWAXS profiles of Htt-0 and Htt-1 monomers (Fig. 3 ▸). We also tried the *SASREF* algorithm of the *ATSAS* package (Cowieson *et al.*, 2020 ▸) for a rigid body refinement with two Htt-1 monomers, based on a whole-helix structure of Htt-1 built from *I-TASSER*. However, the best-fitted model [shown in Fig. 3 ▸(*b*)] also could not describe the helix–helix correlation hump at *q* ≃ 0.45 Å^−1^. Nevertheless, the formation of helical Htt-1 dimers revealed from CD and SEC-SWAXS (*I*_0_ and optical data) suggests an association mechanism of the polyQ peptides through interactions of helical segments.

The low-*q* data of Htt-0 [Fig. 3 ▸(*a*)] deviate slightly, ∼10% lower in intensity, from the fitting profile, which may suggest an effect of interparticle interactions. However, the corresponding UV–Vis intensity and *R*_g_ value evolutions across the SEC-SWAXS elution peak (Fig. S5) demonstrate relatively stable *R*_g_ values, with small fluctuations within 1–2 Å range, despite varying sample concentrations. This consistency suggests negligible interparticle effects in our data fitting, as further shown by the moderate χ^2^ value of 1.9.

Previous ITC results (Tsai *et al.*, 2023 ▸) indicate that the binding affinity of SERF1a to NT17 alone is fourfold of that with Htt-3 and it has little or no binding affinity with Htt-0 or Htt-1. Combining the above structural results for NT17 and the NT17-polyQ of different α-helical contents (Fig. S2), we suggest that the helical conformation of the polyQ region could affect NT17 for adopting helical conformation. Moreover, the highly helical conformation of the NT17-polyQ peptide Htt-1 favors self-association into dimer conformation, compared with interaction with SERF1a. Previous studies have already shown that polyQ peptides could influence the NT17 structure in certain conditions, thereby altering the aggregation behavior of the NT17-polyQ (Thakur *et al.*, 2009 ▸; Urbanek *et al.*, 2020 ▸). The feature of NT17 conformation-dependent interaction with SERF1a revealed here suggests a possible catalytic role for the latter in its facilitating aggregation of Httex1. Presumably, when the polyQ segment of Httex1 begins to form an α-helical structure through SERF1a-facilitated aggregation, this helical configuration could encourage a similar helical transformation in NT17, promoting self-aggregation and thereby reducing interactions with SERF1a. Namely, the increased helical structure within polyQ enhances its self-aggregation, consequently diminishing interactions with SERF1a. Indeed, a previous report by Tsai *et al.* (2023 ▸) supports this hypothesis, indicating minimal presence of SERF1a in the Httex1 fibril aggregates mediated by SERF1a, which underscores its proposed catalytic role.

### 3.3. Complexing of SERF1a with NT17

**Figure 4:**
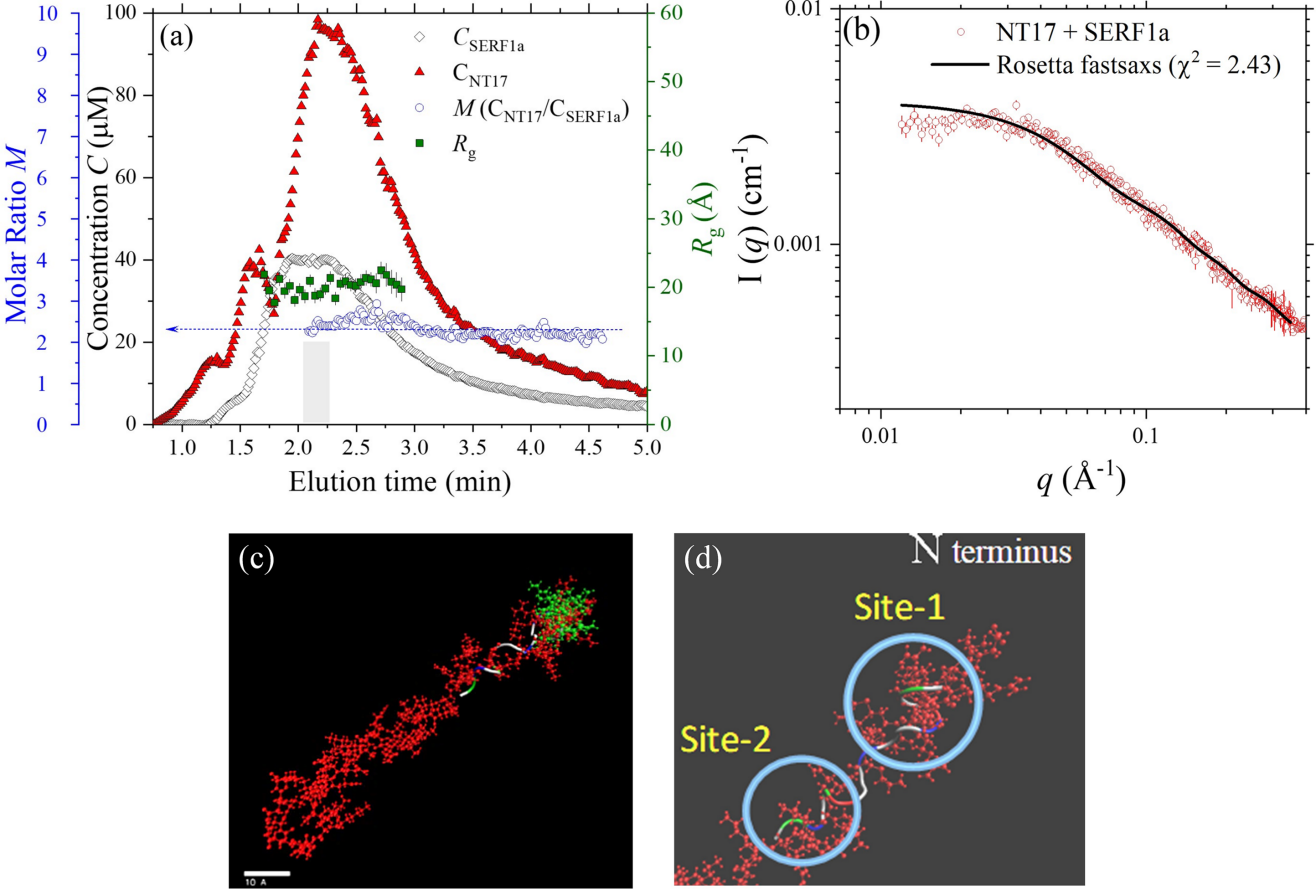
(*a*) Evolutions of *R*_g_, individual concentrations *C* of SERF1a and NT17, and molar ratio *M*, deduced from the SEC-SWAXS elution of the mixture. (*b*) The SWAXS data of the NT17–SERF1a complex (SASDVR5), merged from the data frames shadowed in (*a*), are fitted using *Rosetta-fastsaxs* with the model shown in (*c*). (*c*) The representative *Rosetta-fastsaxs* model with two NT17 (represented by a ribbon and green dots) binding to the N-terminal side of SERF1a (in red). (*d*) A zoom-in view for the binding of the N side of NT17 (colored coil) on the short helical segment (Site-1) and the long coil region (Site-2) on the N-terminal side of SERF1a (*cf*. Table 1 ▸).

In the following, we focus on two complex structures of SERF1a – with NT17 and with Htt-3. Fig. 4 ▸(*a*) shows the evolutions of *R*_g_ (*ca*. 20 Å) and individual concentrations of NT17 and SERF1a of the complex determined from the optical data of UV–Vis absorption and dRI collected over the SEC-SWAXS elution. The result suggests a stable concentration ratio *M* of [NT17]/[SERF1a] ≃ 2 over the elution. Following the method detailed previously, we deduce the aggregation numbers of the complex to be two NT17 with one SERF1a from the *I*_0_ value and the sample component concentrations; this composition is consistent with the binding ratio 2:1 determined from ITC (Tsai *et al.*, 2023 ▸). On the basis of the composition analysis, the SWAXS data of the complex are fitted with an initial model of two NT17 placed close on the N-terminal side of SERF1a in the *Rosetta-fastsaxs* protocol. This spatial arrangement is inspired by the previous NMR result that SERF1a interacts with Httex1-39Q mainly with its helical regions (Tsai *et al.*, 2023 ▸).

Shown in Fig. 4 ▸(*b*) are the SAXS data fitted using the model shown in Fig. 4 ▸(*c*). The model has a relatively minimized free-energy score and a reasonably small χ^2^ value of 2.4 among the ten models constructed by ten independent runs of *Rosetta-fastsaxs* (Fig. S3). The model illustrates that one NT17 tightly entangles with the N terminus of SERF1a, while the other more extended NT17 associates (via its first five amino acids Met1 to Glu5, and C-terminal Leu14 and Phe17) with the short helical segments of Arg7 to Arg11 near the N terminus [Site-1 in Fig. 4 ▸(*d*)] and Thr32 and Arg36 in the central coil region [Site-2 in Fig. 4 ▸(*d*)] of SERF1a. Although the local structural features proposed by the *Rosetta* model may not be unique, the *Rosetta* model (of complementary optimized free-energy scores and SWAXS data fitting χ^2^) could elucidate a reliable global complex conformation and likely local structural features of the SERF1a–NT17 complex as a basis for further structural verification. We observe a minor interparticle effect in the low-*q* data [Fig. 4 ▸(*b*)], indicated by a slight (10–15%) intensity deviation from the fitting profile. This effect may contribute to the minor fluctuations observed in the composition profile [see Fig. 4 ▸(*a*)], which is derived from the concentration-normalized zero-angle scattering intensity, *I*(0)/*C*, throughout the SEC-SWAXS elution.

**Figure 5:**
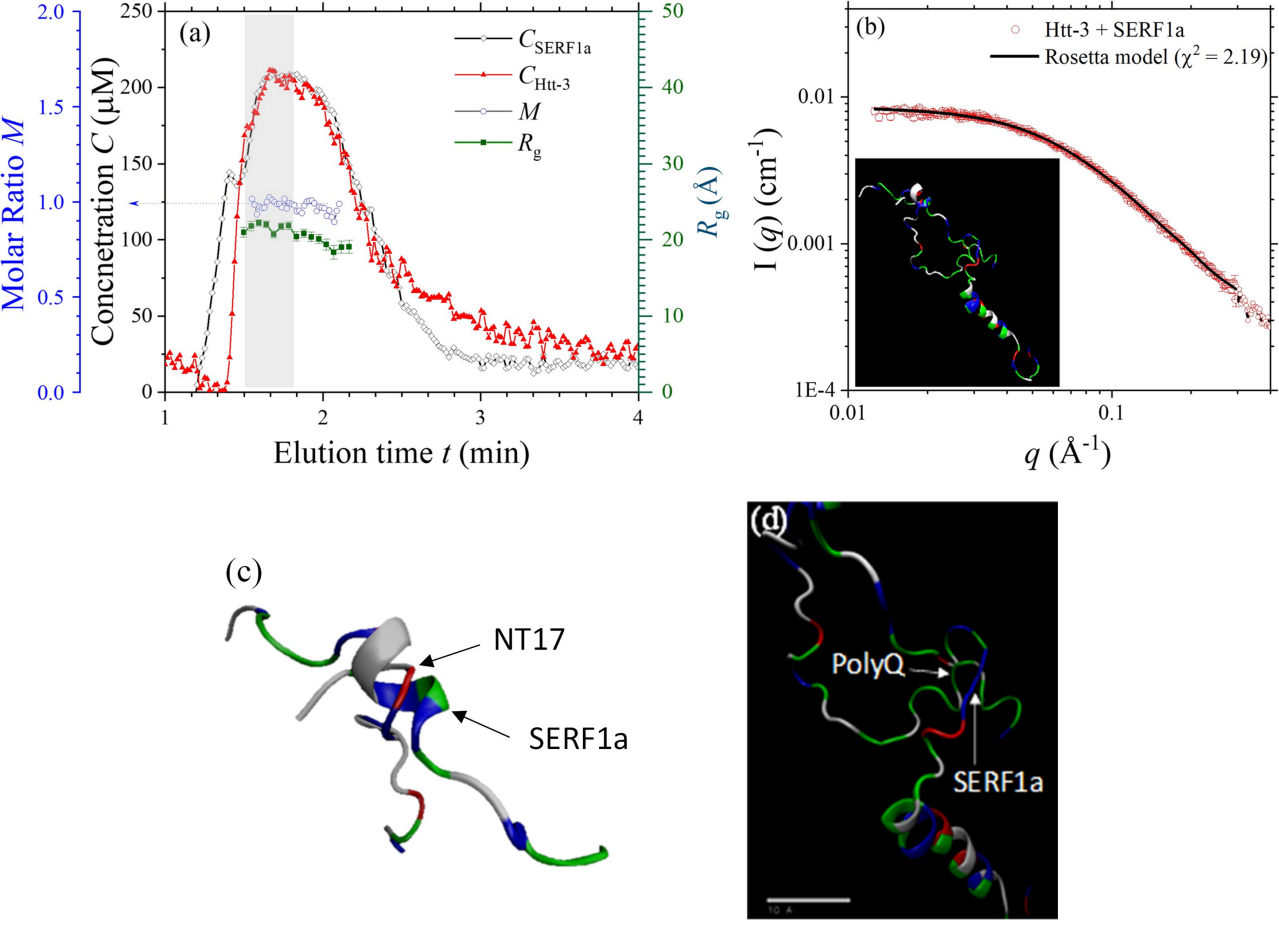
(*a*) Evolutions of *R*_g_, decomposed concentrations of SERF1a and Htt-3 in µ*M*, and the molar ratio *M* of the complex of Htt-3/SERF1a, over the SEC-SAXS elution peak. (*b*) The SEC-SAXS data, merged from the data frames shadowed in (*a*), are fitted (solid curve with χ^2^ = 2.19) using a representative *Rosetta* model (inset; SASDVS5) comprising one SERF1a and one Htt-3. Furthermore, (*c*) and (*d*) are zoom-in views of the two binding sites of Htt-3 (via Thr3 and Pro28 of the NT17-polyQ) with the N-terminal side (Asn5 and Lys23 amino acids) of SERF1a.

Parallel analysis of the SEC-SWAXS result for the complex of SERF1a with Htt-3 is shown in Fig. 5 ▸, with the evolutions of *R*_g_ and decomposed concentrations of Htt-3 and SERF1a [Fig. 5 ▸(*a*)] of the complex over the SEC-SWAXS elution. The molar ratio *M* of Htt-3 over SERF1a over the elution peak is ∼1. Determined from the absolute intensity *I*_0_ and the sample concentration, the complex comprises one NT17 and SERF1a for a 1:1 binding ratio. This binding ratio is significantly lower than the 1:2 binding ratio with NT17 segments alone. Consistently, previous ITC results also revealed a significantly lower binding affinity of SERF1a–Htt-3 than that of SERF1a–NT17, but still with a binding ratio of 2:1 for Htt-3:SERF1a (Tsai *et al.*, 2023 ▸). The lower binding ratio of 1:1 deduced here might be attributed to a fast sample mixing process used in the SEC-SWAXS measurement, compared with the slow Htt-3 titration process into the SERF1a solution in the ITC measurement. Nevertheless, according to the 1:1 binding ratio deduced from the SEC-SWAXS sample elution, the SWAXS data were fitted with an initial model of one Htt-3 with one SERF1a in the *Rosetta-fastsaxs* fitting protocol. Shown in Fig. 5 ▸(*b*) are the SWAXS data fitted with an optimized model, with relatively minimized free-energy score and χ^2^ = 2.19 (Fig. S4). The model reveals two major interactions sites of Thr3 (NT17 segment) and Pro28 (polyQ segment) of Htt-3 with Asn5 and Lys23 of the coil segments of SERF1a, respectively, via hydrogen bonding. Furthermore, the model suggests that although the long helical segments and the nearby amino residues of SERF1a do not directly interact with Htt-3, the conformation is also influenced, which is consistent with the NMR observation reported previously (Tsai *et al.*, 2023 ▸). The local interaction sites are determined mainly by minimization of the *Rosetta* free-energy score and would fluctuate somewhat over the models of comparable free-energy scores and χ^2^ (as shown in Fig. S4). However, these models collectively propose that the NT17-polyQ of Htt-3 loosely attaches to the N-terminal side of SERF1a, via only a few binding sites. This result is consistent with the significantly weaker interactions revealed by ITC, when compared with the robust binding observed between the short NT17 segments alone and SERF1a (*cf*. Fig. 4 ▸).

### 3.4. Model uncertainty and heterogeneities

We have developed representative models for the non-compact SERF1a, NT17 and NT17-polyQ in solution using size-exclusion SWAXS instrumentation, combined with data analysis from SWAXS, NMR, optical spectroscopy and molecular simulation. These models incorporate all relevant structural features. In our analysis of the SWAXS data, we assumed a single conformation based on selecting data with the same *R*_g_ value from the SEC-SWAXS elution profile (refer to Fig. S5). However, these models exhibit certain local structural uncertainties, as indicated by the non-zero values of the NSD, shown in Table 2 ▸. The NSD values were calculated from each set of the ten best-fitted models with comparable free-energy scores and χ^2^ values. Such uncertainties, primarily in local structures, are partly due to the limited *q* range available in the SWAXS data. Furthermore, the current SEC-SWAXS measurement resolution, with frame times of 0.5 or 1 frame per second, does not allow for the differentiation of conformational fluctuations within this time resolution. Expanding the *q* range in SWAXS data and improving the time resolution in SEC-SWAXS data collection are recommended to reduce structural uncertainties and achieve better model convergence in SWAXS-directed molecular dynamics simulations (Hub, 2018 ▸; Manalastas-Cantos *et al.*, 2021 ▸; Sønderby *et al.*, 2017 ▸).

Beyond modeling uncertainties, the intrinsic structural ensembles of the SERF1a–polyQ complex are revealed through their SEC-SWAXS elution profiles, which show varying *R*_g_ values [see Figs. 4 ▸(*a*) and 5 ▸(*a*)]. In this study, we have selectively analyzed data from a finite range of single *R*_g_ values. Data showing slowly changing *R*_g_ values along the elution profiles, which indicate stable compositions and reveal conformational polymorphism, were not analyzed. However, the minor changes in *R*_g_ suggest that conformational inhomogeneities are not significant relative to the representative conformations derived from the data of stable *R*_g_ regions. We propose that conformational ensembles of a non-compact biomolecule are better represented using a complete set of models constructed from the corresponding SEC-SWAXS data with progressively changing *R*_g_ values along the elution profile. In such analyses, maintaining a constant *I*(0)/*C* value (concentration-normalized zero-angle scattering intensity) is crucial to ensure there is no aggregation or decomposition, as demonstrated by the composition profiles in Figs. 4 ▸(*a*) and 5 ▸(*a*). The advantages of using SEC-SWAXS in conjunction with concurrent measurements of UV–Vis light absorption and dRI are particularly evident when analyzing the conformation ensembles of non-compact biomolecules in solution.

## 4. Conclusions

In this investigation, we establish a comprehensive protocol for SEC-SWAXS data analysis, integrating methodologies from *I-TASSER*, *CRYSOL* and *Rosetta-fastsaxs*. This approach enables the construction of molecular models for the coiled protein and peptides of SERF1a, NT17 fragments, NT17-polyQ peptides, and their complexes. The study further highlights the synergistic benefits of integrating UV–Vis absorption and dRI data with SAXS absolute scattering intensity to determine the composition of the complexes. Accurate composition determination is established as a crucial prerequisite for constructing binding structural models, such as SERF1a with two NT17 fragments and one Htt-3. The molecular models generated through this protocol encapsulate all structural features disclosed by complementary SWAXS and NMR data, supplemented by reduced free-energy considerations, although they may not represent ultimate structures. Consequently, these models serve as valuable references for understanding the global and local structures of coiled proteins and peptides in solution. Based on these models, the study reveals that NT17 fragments exhibit robust binding to both the coil and helical segments on the N-terminal side of SERF1a. Interactions between NT17 and SERF1a diminish as the helical content increases in the NT17-polyQ peptides. This conformation-dependent binding affinity suggests a dynamic association of SERF1a with Httext1 during fibrillization, involving a disorder-to-order transition of the polyQ segments from coil to α-helix.

## 5. Related literature

The following references are only cited in the supporting information for this article: Goddard & Kneller (2001 ▸).

## Supplementary Material

Supporting information. DOI: 10.1107/S2052252524006341/ti5031sup1.pdf

Supporting video. DOI: 10.1107/S2052252524006341/ti5031sup2.mp4

SASBDB reference: SERF1a, SASDVL5

SASBDB reference: Htt-3, SASDVM5

SASBDB reference: NT17, SASDVN5

SASBDB reference: Htt-0, SASDVP5

SASBDB reference: Htt-1, SASDVQ5

SASBDB reference: NT17–SERF1a complex, SASDVR5

SASBDB reference: Htt-3–SERF1a complex, SASDVS5

## Figures and Tables

**Table 1:** The sequence of amino acids of NT17 and NT17-polyQ peptides of Htt-0, Htt-1 and Htt-3 The amino acids of SERF1a are marked in bold for the helical segments determined by NMR backbone assignments.

Sample	Sequence
NT17	MATLEKLMKAFESLKSF
Htt-0	MATLEKLMKAFESLKSFQQQQQQQQQQQQQQYK
Htt-1	MATLEKLMKAFESLKSF*L*QQ*L*QQQ*L*QQ*L*QQQYK
Htt-3	MATLEKLMKAFESLKSF*P*QQ*P*QQQ*P*QQ*P*QQQYK
SERF1a	MARGNQR**ELARQ**KNMKKTQEISKGKRKEDSLT**ASQRKQRDSEIMQEKQKAA**NEKKSMQTREK
	1------8---12-------------------33----------------51---------62

**Table 2:** NSD from ten best-fitted models (of comparable χ^2^) of independent runs of the *Rosetta-fastsaxs* modeling for the samples indicated

Sample	NSD
SERF1a	1.29
NT17	0
Htt-0	0.69
Htt-3	0.41
SERF1a/NT17 (1:2)	0.61
SERF1a/Htt-3 (1:1)	0.63
