# Supplementary material for: Binding structures of SERF1a with NT17-polyQ peptides of huntingtin exon 1 revealed by SEC-SWAXS, NMR and molecular simulation
Source: IUCrJ. 2024 Aug 8;11(Pt 5):849–58. doi: 10.1107/S2052252524006341 (PMC11364024; doi:10.1107/S2052252524006341)
Supplement: Supplementary file 1 [file m-11-00849-sup1.pdf]

IUCrJ

Volume 11 (2024)

Supporting information for article:

Binding structures of SERF1a with NT17-polyQ peptides of Huntingtin exon 1 revealed by SEC-SWAXS, NMR and molecular simulation

Tien-Chang Lin, Orion Shih, Tien-Ying Tsai, Yi-Qi Yeh, Kuei-Fen Liao, Bradley W. Mansel, Ying-Jen Shiu, Chi-Fon Chang, An-Chung Su, Yun-Ru Chen and U-Ser Jeng

S1. Physical parameters of SERF1a and the polyQ peptides

Table S1 Molar mass M_w, UV-vis molar absorption coefficients ε_{214} at 214 nm, and dn/dc values of the samples studied.

Sample	M_w (Da)	ε_{214} (M^{-1} cm^{-1})	dn/dc (mL/g)
SERF1a	7336.3	64310	0.1859
NT17	1974.4	27715	0.1979
HTT0	4060	49887	0.1731
HTT1	3999	49499	0.1975
HTT3	3935	60019	0.1946

S2. Rosetta-fastsaxs modeling with SERF1a

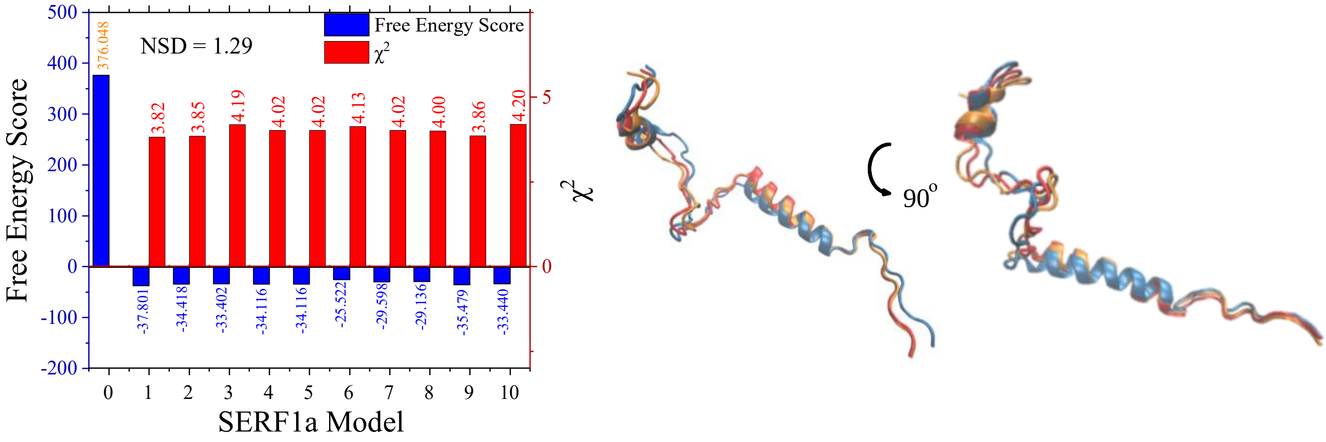

Figure S1 Free energy scores and fitting χ^2 values of the ten models form 10 independent runs of the Rosetta-fastsaxs on the SWAXS data of SERF1a, with the NMR data constraints. Model 0 is the initial model before the Rosetta-fastsaxs refinement. Model 1 is selected as a representative refined model. The value of the normalized spatial discrepancy (NSD) of the 10 models is 1.29. To the right-hand-side are the two orthogonal views of the alignment of the three models #1, #2, and #9 of nearly the same χ^2 values, using CIFSUP of ATSAS (Manalastas-Cantos et al., 2021); the corresponding normalized spatial discrepancy (NSD) value is 1.18, contributed mainly by the relatively fluctuated terminal coils and loops between the helical sections, as illustrated.

S3. CD results

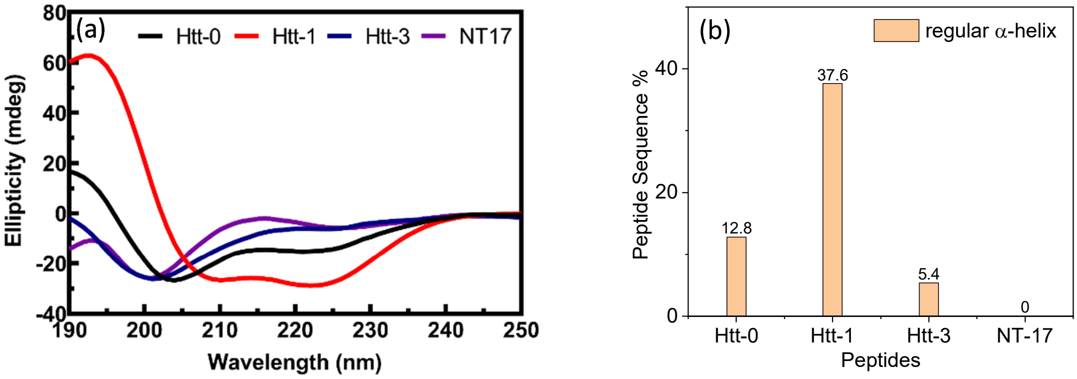

Figure S2 (a) CD spectra of the NT17, Htt-3, Htt-0, and Htt-1, exhibiting successively enhanced α-helical features, characterized by the two minima at 222 and 208 nm and one maximum at 193 nm. (b) The relative α-helix contents (in terms of peptide sequence %) extracted from the CD profiles.

S4. Rosetta modelling with SWAXS data fitting of SERF1a/NT17 and SERF1a/Htt-3 complex

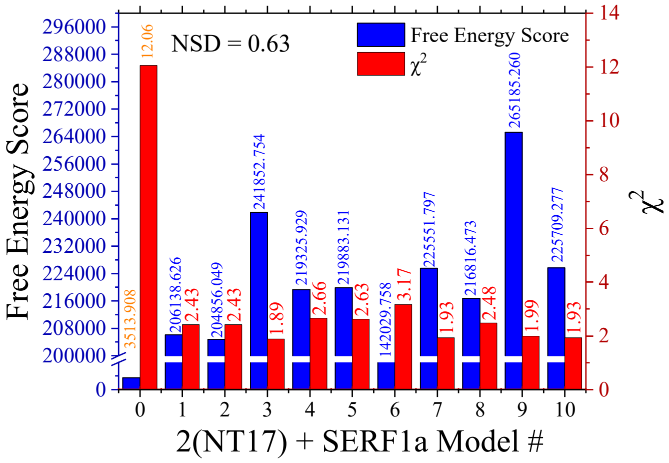

Figure S3 Free energy scores and fitting χ^2 values of 10 independent runs of the Rosetta-fastsaxs of the SWAXS data of the complex of SERF1a with two NT17. The 2nd run with $\chi^2 = 2.43$ and relatively lower free energy score (Model #2) is selected as a representative model.

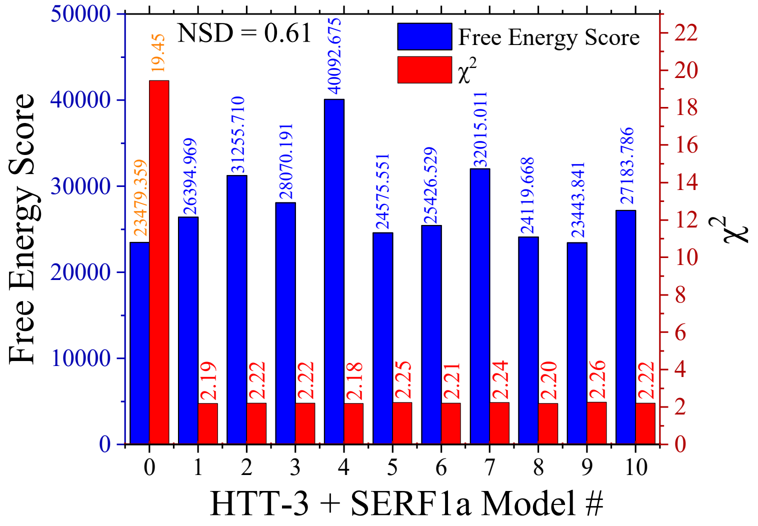

Figure S4 Free energy scores and fitting χ^2 values of 10 independent runs of the Rosetta-fastsaxs of the SWAXS data of the complex of SERF1a with one Htt-3. The Model #1 (1st run) with the smallest χ^2 = 2.19 and a relatively lower free energy score is selected as a representative model.

S5. Full SEC elution profiles of UV and SAXS I_o and R_g

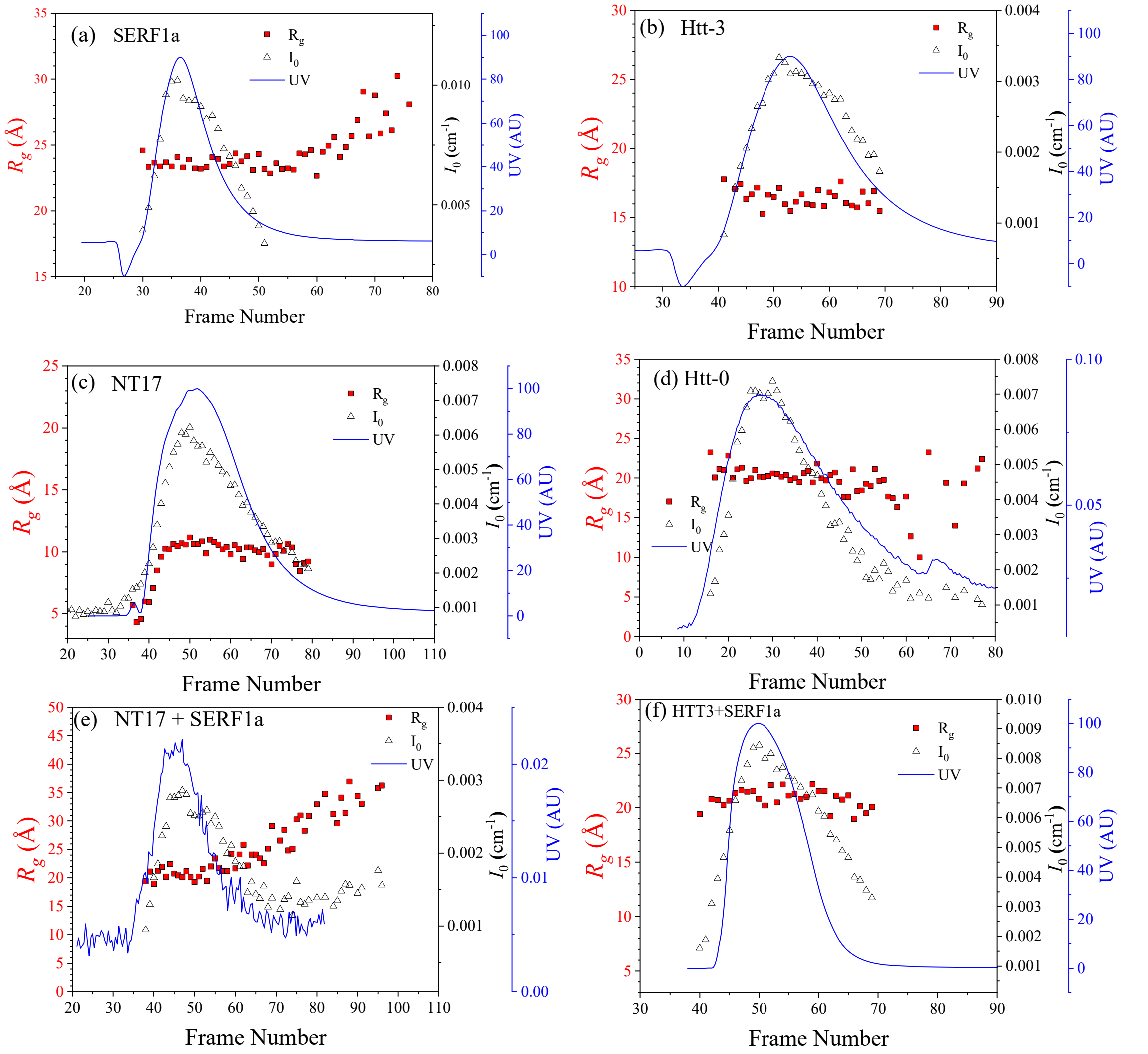

Figure S5 Full SEC elution profiles of UV and SAXS I_o and R_g for SERF1a, N17, and the polyQ peptides as indicated. Note that the UV profiles were measured with the in-line HPLC unit of the SEC-SWAXS elutions. Note that the R_g values determined beyond the elution peaks using the Guinier approximation are of larger deviations due to the decreased I_o values with diminishing concentrations (cf. the UV signals).

S6. NMR models

Due to the lack of NOE information, we could not determine high resolution NMR structure for SERF1a. Therefore, rather than discussing structure details, NMR was applied to identify binding site in our previous paper (Tsai, 2023). In this manuscript, 10,000 NMR structure models of SERF1a were built by Chemical-Shift-Rosetta (CS-Rosetta) (Lange, O. et al. 2012) using the NMR backbone

chemical shift assignment on BMRB CS-Rosetta Server (https://csrosetta.bmrb.io/submit). SERF1a NMR sequence specific backbone chemical shifts were assigned from analysis of HNCA, HN(CO)CA, HNCO, HN(CA)CO, CBCA(CO)NH, and HNCACB spectra. All NMR experiments were conducted at 298K on Bruker Avance 600MHz NMR spectrometer equipped with 5 mm triple resonance cryoprobe with Z-axis gradient. ^{1}H chemical shifts were externally referenced to 0 ppm methyl resonance of the 2,2-dimthyl-2-silapentane-5-sulfonate (DSS), and ^{13}C/^{15}N chemical shifts were indirectly referenced according to the IUPAC recommendations as described previously (Tsai, et al. 2023). NMR data were collected and processed using software Topspin2.1 (Bruker, Germany) and further analyzed by Sparky version 3.114 (Goddard and Kneller).

Table S2. SAXS data collection parameters and processing details

Sample name	SERF1a	Htt-3	NT17	Htt-0	Htt-1	NT17+SERF1a	Htt-3+ SERF1a
(a) Sample details							
Organism	Homo sapiens	Synthetic	Synthetic	Synthetic	Synthetic	Synthetic	Synthetic
UniProt sequence ID (residues in construct)	O75920-2	N/A	N/A	N/A	N/A	N/A	N/A
Calculated molecular weight M_w	7.34 kDa	3.89 kDa	1.97 kDa	4.01 kDa	3.95 kDa	11.28 kDa	11.23 kDa
SEC column	NA (bypass column)			Agilent Bio SEC-3 LC Column (pore size 300 Å)	NA (bypass column)		
Number of frames used for data analysis	12	5	5	10	3	6	5
Injected volume (μL)	50	100	80	70	100	60	100
Loading concentration (mg/mL)	4.1	10	1.6	10	10	0.445 (NT17) 0.255 (SERF1a)	0.8 (Htt-3) 0.7 (SERF1a)

Flow rate (mL/min)	0.1	0.1	0.1	0.35	0.5	0.1	0.1
SEC buffer	Sodium phosphate buffer (PB) solution (containing 480 µL of 10 mM PB, pH 7.4, 16.5 µL of 100 mM NaOH, and 10 µL of 1% TFA)						
(b) SAXS data collection parameters							
Instrument	TPS 13A BioSWAXS beamline of the National Synchrotron Radiation Research Center[1,2]						
Wavelength (Å)	0.8265						
q range ($Å^{-1}$)	0.007 – 0.6 (SAXS); 0.4 – 2.4 (WAXS)						
Sample-to-detector distance (m)	2.5 (Eiger X 9M) 0.288 (Eiger X 1M)						
Exposure frame time	2 sec/frame						
Temperature (°C)	10						
Detector(s)	Eiger X 9M (SAXS) and X 1M (WAXS) detectors, both in vacuum						
Flux (photons/s)	$\sim 1\times10^{12}$						
Beam size (µm)	300(H)x225(V)						
Sample configuration	2.0 mm diameter quartz capillary						
Absolute scaling method	Scaling to absolute water scattering intensity						
Normalization	Accumulated monitor counts of the incident X-ray flux over the sample measuring time.						
(c) Structural parameters							
R_g (Å) [from $p(r)$]	24.1±0.1	20.1±0.1	12.1±0.1	20.3±0.1	19.1±0.1	23.1±0.1	23.0±0.1
R_g (Å) [from Guinier Approximation]	24.0±0.1	20.0±0.1	12.0±0.1	20.3±0.1	19.0±0.1	23.0±0.1	22.9±0.1
D_{max} (Å)	89.0	78.4	41.7	61.8	75.8	83.9	85.1

M_W determined from size & shape (kDa). (only for compact proteins)	9.63	2.16	1.37	9.55	17.7	5.00	5.47
M_W determined from V_c (kDa) (only for compact proteins)	7.25	1.86	2.37	10.03	16.1	3.08	3.73
(d) Software employed							
SAXS data reduction and data processing	TPS 13A SWAXS Data Reduction Kit (Ver. 4.88) [1]<br>PRIMUS (ATSAS 3.2.1)						
Computation of model intensities	CRYSOL (ATSAS 3.2.1)						

Reference

1. Shih, O.; Liao, K.-F.; Yeh, Y.-Q.; Su, C.-J.; Wang, C.-A.; Chang, J.-W.; Wu, W.-R.; Liang, C.-C.; Lin, C.-Y.; Lee, T.-H.; Chang, C.-H.; Chiang, L.-C.; Chang, C.-F.; Liu, D.-G.; Lee, M.-H.; Liu, C.-Y.; Hsu, T.-W.; Mansel, B.; Ho, M.-C.; Shu, C.-Y.; Lee, F.; Yen, E.; Lin, T.-C.; Jeng, U., *J. Appl. Cryst.* **2022**, 55, 340-352.
2. Liu, D.-G.; Chang, C.-H.; Chiang, L.-C.; Lee, M.-H.; Chang, C.-F.; Lin, C.-Y.; Liang, C.-C.; Lee, T.-H.; Lin, S.-W.; Liu, C.-Y.; Hwang, C.-S.; Huang, J.-C.; Kuan, C.-K.; Wang, H.-S.; Liu, Y.-C.; Tseng, F.-H.; Chuang, J.-Y.; Liao, W.-R.; Li, H.-C.; Su, C.-J.; Liao, K.-F.; Yeh, Y.-Q.; Shih, O.; Wu, W.-R.; Wang, C.-A.; Jeng, U., *J. Synchrotron Rad.* **2021**, 28, 1954-1965
